# Sex-Specific Habitat Utilization and Differential Breeding Investments in Christmas Island Frigatebirds throughout the Breeding Cycle

**DOI:** 10.1371/journal.pone.0129437

**Published:** 2015-06-22

**Authors:** Janos C. Hennicke, David J. James, Henri Weimerskirch

**Affiliations:** 1 Marine Top Predator Group, Centre d’Etudes Biologiques de Chizé, CEBC–CNRS, Villiers-en-Bois, France; 2 Department of Ecology and Conservation, University of Hamburg, Hamburg, Germany; 3 73 Pozieres Ave, Milperra, New South Wales, Australia; Hawaii Pacific University, UNITED STATES

## Abstract

In seabirds, equal bi-parental care is the rule, as it is considered crucial for raising chicks successfully because seabirds forage in an environment with unpredictable and highly variable food supply. Frigatebirds forage in poor tropical waters, yet males reduce and even stop parental care soon after chick brooding, leaving the female to provision the chick alone for an extended fledging period. Using bird-borne tracking devices, male and female Christmas Island Frigatebirds (*Fregata andrewsi*) were investigated during the brooding, late chick rearing and post-fledging period to examine whether sexes exhibit foraging strategies that may be linked to differential breeding investments. During brooding, males and females showed similar foraging behaviour under average marine productivity of oceanic waters close to the colony, but males shifted to more distant and more productive habitats when conditions deteriorated to continue with reduced chick provisioning. During the late chick rearing period, females progressively increased their foraging range to the more distant but productive marine areas that only males had visited during brooding. Birds spent the non-breeding period roosting in highly productive waters of the Sunda Shelf. The sex-specific utilisation of three different foraging habitats with different primary productivity (oceanic, coastal, and shelf areas) allowed for temporal and spatial segregation in the exploitation of favourable habitats which seems to enable each sex to optimise its foraging profitability. In addition, post-fledging foraging movements of females suggest a biennial breeding cycle, while limited information on males suggests the possibility of an annual breeding cycle.

## Introduction

In most bird species, care by both parents is required to rear offspring successfully [1]. This situation is even more general in seabirds where equal biparental care is found in almost all species [2,3,4], probably because seabirds forage at sea where prey availability is often highly variable and unpredictable [5]. Moreover, sexual dimorphism in size is usually small in seabirds, and if differences exist, males are generally larger than females [2,3,6,7].

Frigatebirds appear to be an exception to these rules. Although male and female frigatebirds share duties equally during incubation, it is generally assumed that males progressively reduce and at least in some species eventually even stop their involvement in chick rearing, leaving the female alone to provision the chick till fledging [8–14]. One consequence of this is slow chick growth and frigatebirds have exceptionally long chick rearing periods among birds [3,10,13,15]. This extreme sex difference in parental care is thought to be associated with different strategies of males and females to maximise individual life-time reproductive success [13,14]. Another consequence of extended female chick rearing is that the chick provisioning females can breed only every second year if a chick is fledged successfully while the deserting males can potentially breed annually [9,11,13]. In addition, frigatebirds show sexual size dimorphism which is reversed; females can be up to 30% heavier than males [12,15,16]. This reversed size dimorphism might be a consequence of sexual division of labour which selects the sexes differentially according to their specific task they perform, such as differential parental care during breeding [17–19].

With such an extreme case of differentiation between male and female involvement in chick rearing, fundamental questions arise: are the sex differences in breeding involvement related to differences in foraging behaviour and how do female frigatebirds respond to the progressive reduction in their partner’s involvement in chick provisioning, given the importance of biparental care for successful chick rearing in other seabirds? Sex-specific differences in foraging behaviour in seabirds are limited, but exist in some species: they are generally interpreted as the result of sexual segregation for resources in the foraging grounds [20,21], and are not always related to differences in size [22,23]. However, differences in foraging ecology between sexes generally do not seem to have important consequences for their respective involvement in parental care apart from slight differences in offspring provisioning rates [24] or offspring attendance [19]. In the case of frigatebirds, however, because of the extreme case of differential chick rearing involvement between sexes, we assume that differences in foraging behaviours between the sexes reflect their differential involvement in breeding.

There is only limited information available on frigatebird foraging behaviour in general and none on sex-specific differences. Only recently, bio-logging technologies, like satellite transmitters and GPS loggers, opened up the possibility to investigate the birds’ at-sea behaviour in detail. The best information available is for Great Frigatebird (*Fregata minor*), which exploits oceanic waters and makes use of oceanographic features like eddies and fronts to increase its foraging efficiency [25–28]. In contrast, the Magnificent Frigatebird (*F*. *magnificens*) seems to forage in more costal waters, often in the relative vicinity of its breeding colonies [12,26].

The Christmas Island Frigatebird (*F*. *andrewsi*; hereafter CIFB) is endemic to Christmas Island (hereafter CI) in the eastern Indian Ocean. As in all frigatebird species, CIFB exhibits reversed sexual dimorphism, with females being up to 28% heavier, and extended chick rearing, with chicks being provisioned for up to 15 months after hatching [3,10]. Using bird-borne tracking devices, the present study investigates sex-specific foraging movements and habitat utilization of CIFB during and after the chick rearing period to examine (1) whether males and females exhibit different foraging behaviours while provisioning their chicks jointly, (2) whether females change their foraging behaviour to cope with the males’ reduction in their involvement in chick rearing, and (3) if sexes show foraging movements elucidating their so far unknown breeding cycles.

## Materials and Methods

### Field site and study animals

The study was carried out on Christmas Island (10° 25’ S, 105° 40’ E), the emergent tip of a submarine mountain rising steeply from the surrounding ocean floor of 2,000 m depth, approximately 400 km south of Java [29]. Study nests were located in the ‘Golf Course’ breeding colony where CIFB breed in coastal rainforest at heights between 5 and 25 m above the ground. Nests were located from the ground and accessed by tree-climbing. Breeding birds were caught on their nest by hand and brought down to the ground in a bag for measurements and device attachment and/or retrieval. Sex of adults was determined by bill colour and plumage [15]. At deployment and retrieval, birds were weighed to 10 g precision using a spring balance (Super Samson, Salter, USA). After handling, birds were released at the edge of the forest (within 50 m of their nests) and they typically returned to their nests within 5 min and resumed breeding duties. The total time from catch to release was typically about 20 min.

All animals were captured and handled in accordance with the principles and guidelines of the laws on animal welfare and under permits from Parks Australia North, Christmas Island National Park, and the Australian Department of Environment and Water Resources.

### Data collection

The study investigated the foraging behaviour of CIFB during the brooding, late chick rearing and post-breeding periods. The brooding period was defined as that part of the breeding cycle when the chick was being brooded or guarded constantly by at least one parent. The late chick rearing period was defined as the time when the chick was no longer attended by a parent constantly, but was left alone on the nest and visited only briefly for feeding. During this period, from approximately mid July onwards, males progressively reduced provisioning the chick and hence mainly females could be equipped with tracking devices. The post-breeding period was considered to start when the adult bird carrying the device left the island and did not return for more than a month.

Fieldwork to investigate the brooding period was conducted during May and June 2009 and 2010. Birds were equipped with GPS-loggers (GyPSy, Technosmart, Italy, and iGotU GT-120, Mobile Action, Taiwan) and satellite tags (30g battery PTT-100, North Star Science and Technology LLC, USA) (Table 1). All devices were attached to the four central rectrices using Tesa Tape (Beiersdorf, Germany) for periods of about 3–10 days. The mass of any device including the tape was between 1.4–3.0% of the adult mass, considered appropriate for minimizing effects to seabirds [30]. All deployed devices were recovered and none of them failed but in some GPS-loggers data collection stopped before the end of the trip due to battery exhaustion (see also below).

**Table 1:** Deployments of tracking devices on Christmas Island Frigatebirds during the brooding period (for details of manufacturers see text).

year	device	weight	tracking interval	birds equipped	number of trips tracked
2009	GPS (Technosmart)	30g	1 sec	2 females	6
	GPS (Technosmart)	20 g	1 sec	4 males	8
	Sat tag (North Star)	30 g	uplink approx. every 2 h	4 females	12
2010	GPS (Mobile Action)	22 g	10x 5 min, 3x 10 min	8 females	14
	GPS (Mobile Action)	22 g	10x 5 min, 3x 10 min	5 males	8

Fieldwork to investigate the late chick rearing and post-breeding periods was carried out in September 2005, July 2007 and June 2009. Birds were equipped with satellite transmitters (30 g (for females) and 20 g (for males) battery PTT-100, North Star Science and Technology LLC, USA). Two females were equipped in September 2005 three females were equipped in July 2007, and one male was equipped in June 2009. The satellite tags of females were set on a duty cycle of 10 h ON and 10 h OFF, increasing to 48 h OFF after 1 month and to 72 h OFF after 3 months. The tag of the male was set on a duty cycle of 10 h ON and 96 h OFF for the whole time. All transmitters were attached on the birds’ backs using custom made harnesses of Teflon ribbon (Bally Ribbon Mills, USA) with a weak link made of thin leather. Under the tropical conditions of the study region, the leather will get bridle and eventually break to release the devices from the birds. The weight of the tag and harness was between 2.4–2.8% of the females’ body weight, and it was 2.9% of the male’s body weight. Transmitting periods for each individual are shown in Table 2.

**Table 2:** Details of satellite transmitter deployments on Christmas Island Frigatebirds covering the late chick rearing and post-breeding periods (F = female, M = male).

bird (tag ID)	sex	first transmission	last transmission	number of fixes
60397	F	17.09.2005	18.05.2006	818
60396	F	24.09.2005	01.12.2005	384
67658	F	21.07.2007	18.04.2008	890
60348	F	23.07.2007	21.10.2007	504
67659	F	26.07.2007	13.04.2008	880
94135	M	05.06.2009	03.03.2010	321

### Foraging parameters

#### Brooding period

Start and end times of foraging trips were determined from locational data by averaging the time between the last fix on the nest and the first fix at sea and vice versa. Some trips were not completely covered by GPS recordings due to battery exhaustion. Data on those trips were only used in analyses when appropriate, e.g. start time of trip, and sample sizes are given accordingly.

To reject flawed satellite positions, satellite data were filtered using the R package “Argosfilter” which applies a maximum velocity filter [31]. This maximum flight velocity (27.3 m/s input speed for the filtering process) was calculated from the GPS logger data as the average of the maximum flight speeds recorded during the daytime of each trip.

Distances of birds relative to Christmas Island were calculated using spherical trigonometry (arc distance formula, [32]). To calculate the distances travelled during a trip, only trajectories recorded with GPS-loggers were analysed.

A 50% fixed kernel density estimation was used to determine core foraging areas [33,34]. For the kernel analyses. trajectories obtained from satellite transmitters and the 10 min GPS logger (see above) were interpolated linearly to locations every 5 min, assuming a constant flight velocity and direct flight path between location fixes. The resulting positions (5 min intervals for each individual) were pooled for each sex to determine sex-specific areas during this breeding stage for each year. Kernel analyses were conducted with the R package “adehabitatHR” using longitude/latitude data transformed into UTM (Zone 48) positions and *ad hoc* h-values for kernel smoothing [33,34].

#### Late chick rearing and post-breeding periods

The positional data obtained by satellite transmitters during those periods were treated with the velocity filter (see above, [31]). As all transmitters were set on duty cycles, parameters like foraging trip duration and trip start and end could not be determined precisely. Therefore, only the foraging range was directly derived from the positional data. Trip durations were estimated by using the correlation between trip distance and trip duration obtained from GPS data of females during brooding (τ = 0.738, p < 0.001, n = 30; linear regression for calculation: trip duration = -5.41 + 0.26 * maximum distance, r² = 0.736, p < 0.001, n = 30).

To determine foraging range with the available (duty-cycled) satellite data, data were combined into 10-day bouts (= decade) and only the location farthest away from CI for each bird in each decade was used in analyses. Decade 1 begins on July 21^st^, the date of the first transmission of all females equipped with satellite transmitters during the late chick rearing period (see Table 2).

### Marine conditions

To assess the general prey conditions faced by CIFB in their foraging habitats throughout the breeding cycle, chlorophyll *a* concentration was used. This proxy measure for marine productivity has been shown to be an important factor in frigatebirds for selecting foraging areas [25,27,28]. To gain this qualitative insight, chlorophyll *a* information was compiled from NASA, USA, through its GIOVANNI data gateway (http://disc.sci.gsfc.nasa.gov/ giovanni/overview/index.html). Monthly MODIS Aqua data with a spatial resolution of nine km were used to compile chlorophyll *a* averages for the main three marine habitats used by the birds (see results) for the study period (2005–2010): 1) CI waters: An area of 3 x 3 ° with CI in its middle, corresponding approximately to the core foraging area of CIFB females during brooding; 2) Java Head and the southern coast of Java, and 3) the Sunda Shelf between Borneo, Malaysia, Sumatra and Java.

### Statistical analyses

Statistical analyses were performed with SPSS 11.5 (SPSS Inc., Chicago, USA), and R Studio (Version 0.94.92, RStudio, Inc) using the R version 2.13.0 [35].

Normality of the data was checked by Q-Q-plots or, in the case of small sample sizes, with application of the Shapiro-Wilk-Test. Where necessary, appropriate transformations were performed to gain normality, i.e. ln-transformation of trip duration. Heteroscedasticity was checked using plots of residuals over fitted values or, in the case of small sample sizes, with Levene-Tests for heteroscedasticity.

To examine differences in foraging parameters (trip duration, maximum distance from island, distance travelled) within and between sexes and years, Linear Mixed Models with sex, season and their interaction as fixed factors were applied. As data on several foraging trips per individual were analysed, bird identity was included in all models as a random factor to avoid pseudoreplication. The significance for each parameter was determined by *F* statistics.

Kendall’s τ-b Correlation tests were used to examine correlations between trip duration and trip distance during brooding (see above).

During the late chick rearing period, all birds (n = 5) showed the same behaviour independent of the study year (linear regressions for each tracked individual: all r² > 0.359, all p < 0.05, n: 6–21). Thus, data from all individuals were pooled and a linear regression was used to examine the relationship between date (i.e. chick age) and trip distance during late chick rearing.

For all tests, the threshold for significance was p < 0.05 and all tests were 2-tailed. Means are given with ± SD, medians with minimum and maximum values.

## Results

### Brooding period

In total, 32 foraging trips of 14 females and 16 trips of 9 males were recorded during the brooding periods of 2009 and 2010 (Table 1).

**Fig 1:**
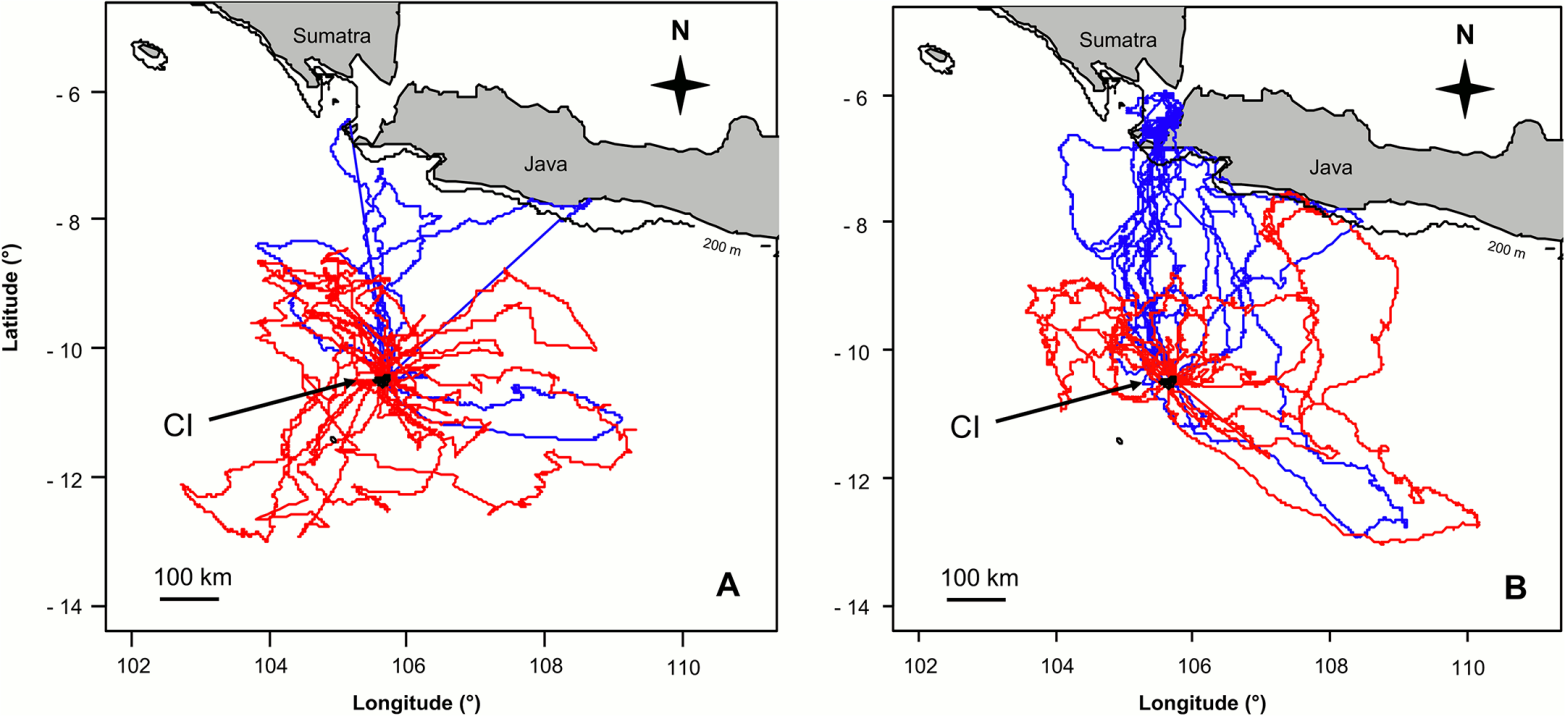
Foraging movements during the brooding period. Foraging tracks of Christmas Island Frigatebirds during the brooding periods of (a) 2009 and (b) 2010 determined by GPS-loggers and satellite transmitters (female tracks = red, male tracks = blue; thin black line = 200 m isobath).

CIFB made long and distant foraging trips (Fig 1, Table 3), with significant sex differences between years in trip duration, maximum distance and distance travelled (F_1, 39_ = 6.108, F_1, 40_ = 6.70, F_1, 27_ = 5.351, respectively, all p < 0.05). While there were no significant differences between males and females in any of the foraging parameters in 2009 (F_1, 39_ = 6.108, F_1, 40_ = 6.70, F_1, 27_ = 5.351, respectively, all p > 0.05), males travelled farther (F_1, 18_ = 8.138, p = 0.011) and covered more distance (F_1, 17_ = 4.4854, p = 0.042) than females in 2010. Trip duration was not significantly different between sexes (F_1, 17_ = 3.864, p = 0.66). There were no significant differences between years in female trip duration, maximum distance and distance travelled (F_1, 28_ = 0.070, F_1, 28_ = 0.945, F_1, 16_ = 0.663, respectively, all p > 0.1), while all three parameters significantly increased in males in 2010 (F_1, 5,334_ = 10.743, F_1, 12_ = 5.677, F_1, 11_ = 7.283, respectively, all p < 0.05).

**Table 3:** Foraging parameters of male and female Christmas Island Frigatebirds during the brooding periods in 2009 and 2010.

year	parameter	females (±SD; n)	males (±SD; n)
2009	trip duration (h)	41.7 ± 29.1 (18)	26.3 ± 23.2 (6)
	max. distance (km)	202.8 ± 107.7 (18)	204.6 ± 161.7 (6)
	distance travelled (km)	999.1 ± 581.1 (6)	634.7 ± 523.4 (6)
2010	trip duration (h)	54.0 ± 52.0 (12)	99.2 ± 60.6 (7)
	max. distance (km)	194.2 ± 162.9 (12)	413.1 ± 116.4 (8)
	distance travelled (km)	963.5 ± 976.0 (6)	1899.7 ± 1056.9 (7)

**Fig 2:**
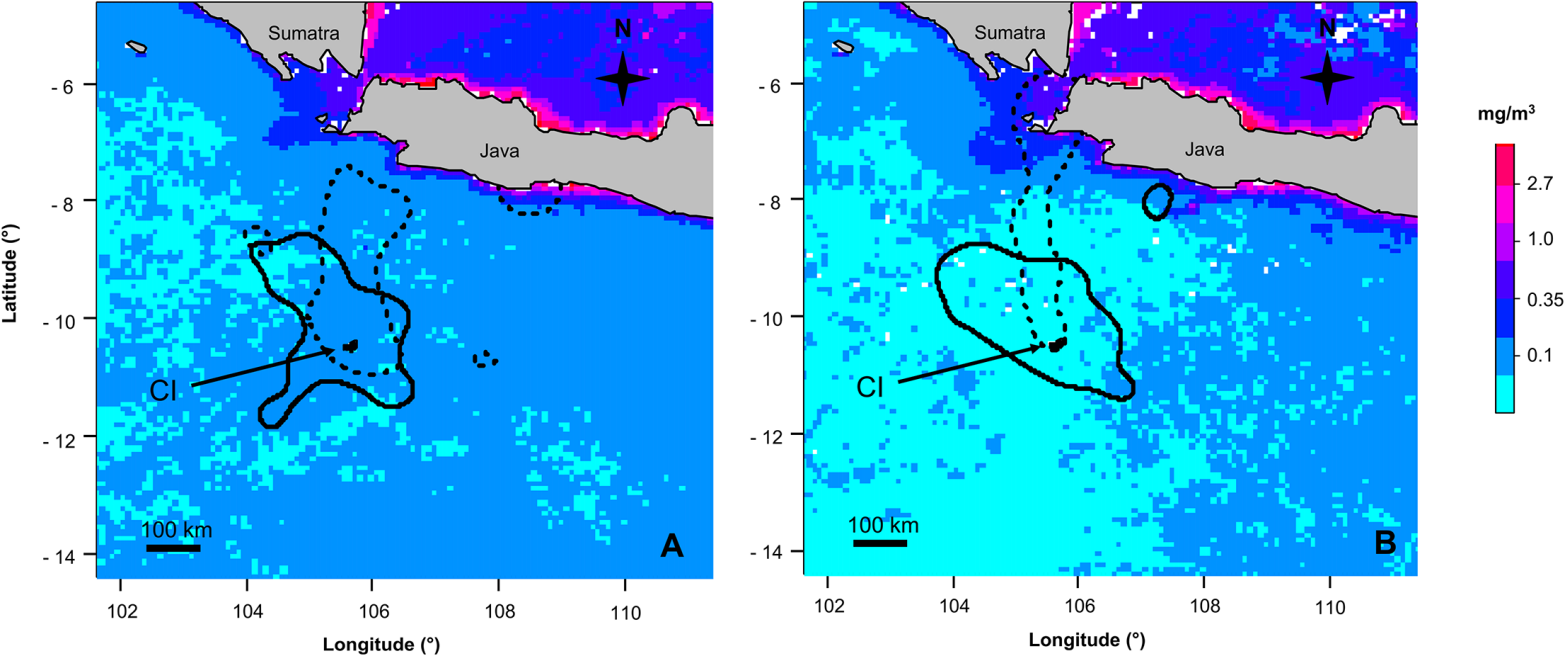
Core foraging areas and chlorophyll *a* concentrations during the brooding period. Core foraging area (50% Kernel density estimation) of Christmas Island Frigatebirds and chlorophyll *a* concentrations during the brooding periods of (a) 2009 and (b) 2010 (females = solid line, males = dashed line; chlorophyll *a* concentration for May and June combined, logarithmic scale).

In both years, the core foraging area of females was an ellipsoid area around Christmas Island (Fig 2). Only one female foraged further north than 8.5° S, getting close to the southern coast of Java, while all other females stayed further south in oceanic waters and did not utilise coastal waters of Java (Fig 1). In contrast, males travelled farther north and also utilised shallow, coastal waters off Java in both years (Figs 1 and 2). Especially in 2010, oceanic waters around CI were hardly utilised but the core foraging area was a small corridor stretching from Christmas Island to Java Head (Fig 2B).

### Late chick rearing period

**Fig 3:**
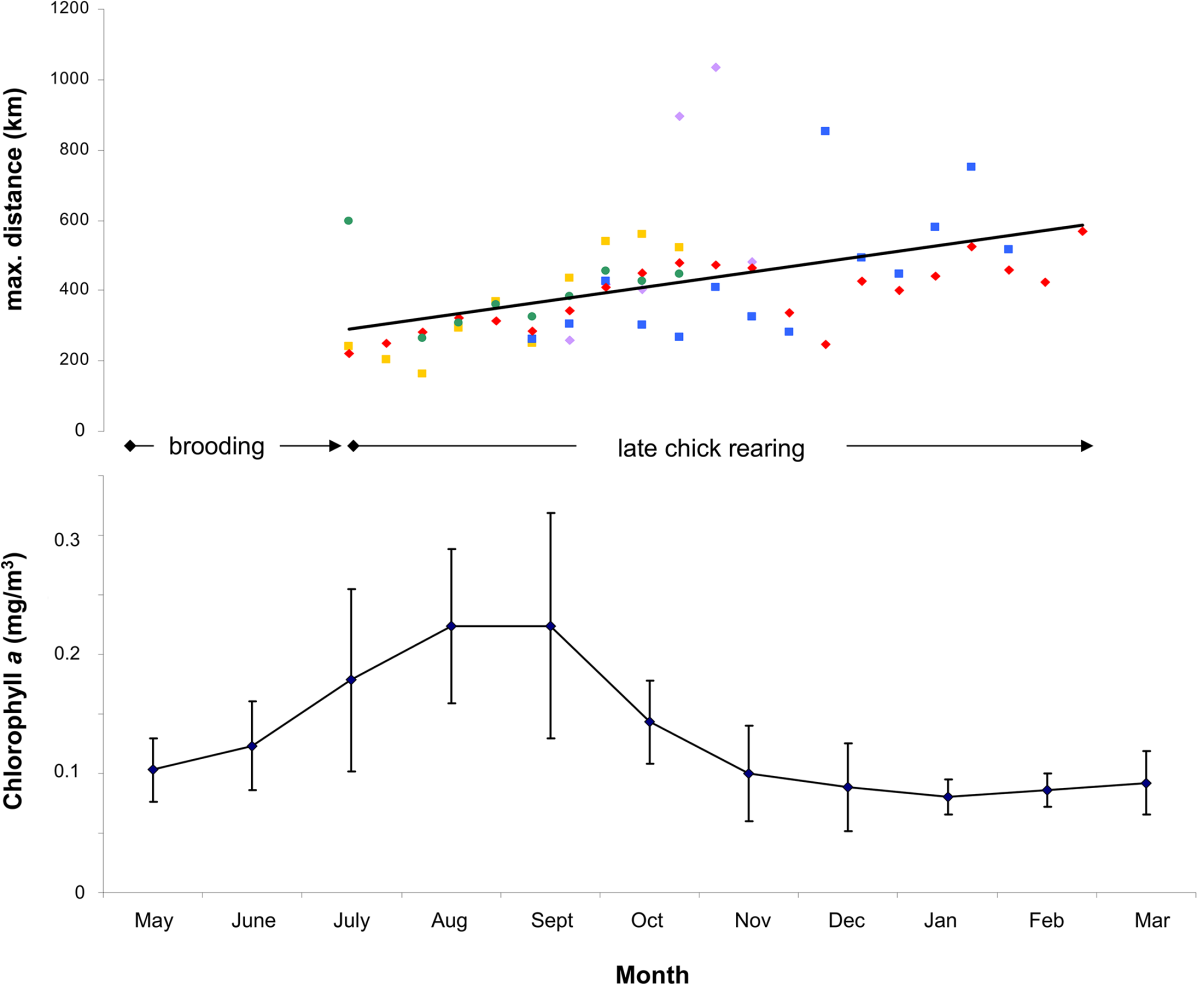
Maximum foraging distances during the late chick rearing period and chlorophyll *a* concentrations around CI. Maximum foraging distance of female Christmas Island Frigatebirds during the late chick rearing period (each individual in a different colour, regression line in black) and chlorophyll *a* concentration around CI (monthly means for study period 2005–2010, with SD).

**Fig 4:**
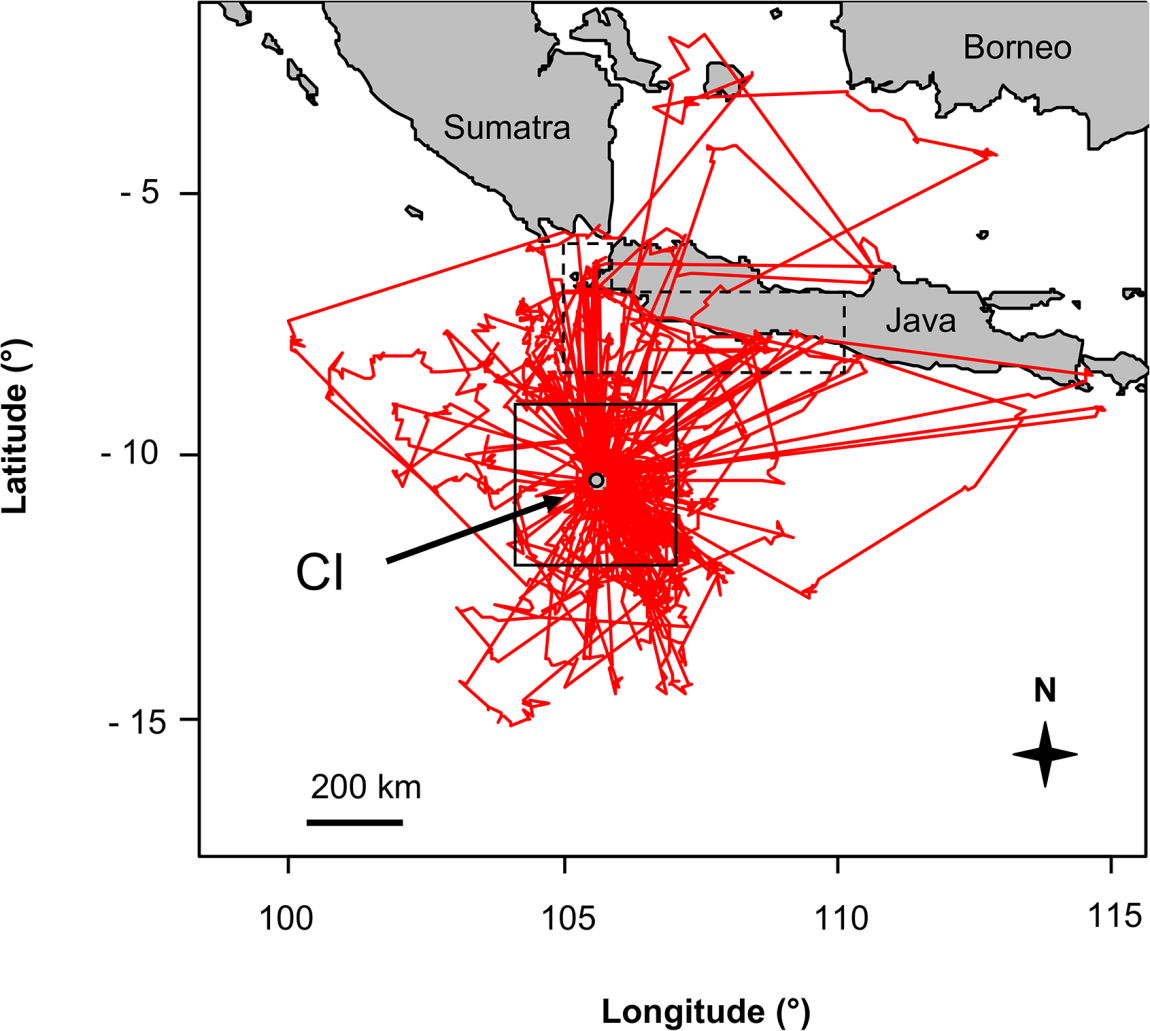
Foraging movements during the late chick rearing period. Tracks of foraging trips of female Christmas Island Frigatebirds during the late chick rearing period determined by satellite telemetry. The solid box delineates the “CI waters area” and the dashed box delineates the “Java Head and South Coast area” for which the chlorophyll *a* data was compiled (see methods).

During late chick rearing, when females provision the chick while males progressively reduce nest attendance, the foraging movements and habitat utilization of females changed: the foraging range increased constantly with date, i.e. chick age (linear regression: r^2^ = 0.220, p < 0.001, n = 60; Fig 3). Females now utilized marine areas that encompassed also coastal areas of Java and waters of the Java Sea that they had not used during brooding (Fig 4).

Calculated trip durations had a median of 98.6 h (range 32.9–266.6 h, n = 60) and hence were on average about twice as long as during the brooding period (Table 3).

### Post-breeding period

**Fig 5:**
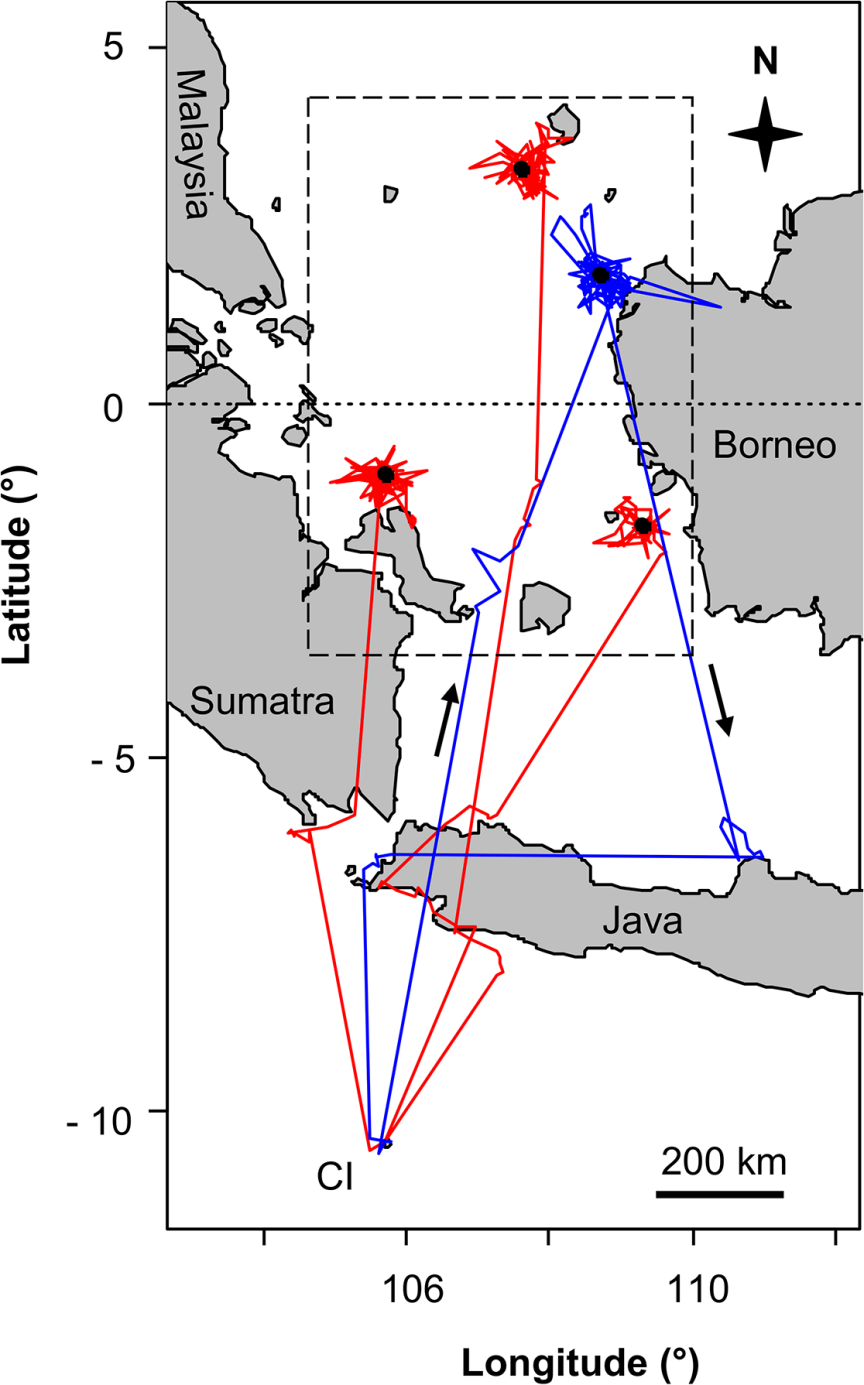
Migration and foraging movements during the post-breeding period. Tracks of Christmas Island Frigatebirds during the post-breeding period determined by satellite telemetry (females = red lines, male = blue line, roosts = black dots, arrows indicate travel direction). The dashed box delineates the “Sunda Shelf area” for which the chlorophyll *a* data was compiled (see methods).

Three females were tracked long enough to record their post-breeding departure from CI. One female left the island at the beginning of November, while the two other females left in February. Upon departure, all three flew north to equatorial waters of the South China Sea and Java Sea over the Sunda Shelf between the Malay Peninsula, Sumatra, Java and Borneo, about 1,000–1,500 km away from CI. They roosted on small islets, remained sedentary and made only relatively short foraging trips (Fig 5). They rarely travelled > 50 km from their roost islands and all daytime locations were at sea and all night time locations were on the island (allowing for some locational inaccuracies), indicating that foraging trips lasted from dawn to dusk. All three birds were still present at their roost islands when their transmissions ceased in mid April (two birds) and mid May (Table 2).

One male was tracked for 9 months from June 2009 until the new breeding season in 2010. He stopped visiting his chick at the end of July, whereas the female continued to provision the chick (checked by observations until October). The male flew north to roost on an islet and foraged in equatorial waters off the west coast of Borneo (Fig 5). He remained there until mid January when travelled back to CI where he stayed until his satellite tag fell of in March.

### Chlorophyll *a* concentrations in main foraging habitats

The three main foraging areas of CIFB showed substantial differences in mean chlorophyll *a* concentrations. Throughout the study period, the Sunda Shelf area had the highest concentrations with 0.82 mg m^-3^ (± 0.285, n = 72 months). The southern Java coast and Java Head had slightly lower concentrations (0.73 mg m^-3^ ± 0.987, n = 72) while the waters around CI had the lowest concentrations with a mean of only 0.13 mg m^-3^ (± 0.069, n = 72) (Fig 2, example for May/June). In May and June, chlorophyll *a* concentrations around CI were low but concentrations increased and peaked in August and September. Values dropped in the subsequent month to reach their lowest levels from November onwards (Fig 5). During the brooding period of 2009, chlorophyll *a* concentrations around CI were about average compared to the whole study period (May: 0.11 ± 0.018 mg m^-3^ vs. 0.10 ± 0.026 mg m^-3^; June: 0.11 ± 0.010 mg m^-3^ vs. 0.12 ± 0.037 mg m^-3^), while in 2010, the concentrations were about 1/3 below average (May: 0.07 ± 0.016 mg m^-3^; June: 0.08 ± 0.010 mg m^-3^) (Fig 2).

## Discussion

This is the first study to collect data on sex-specific foraging movements and habitat utilization of a frigatebird species covering the brooding, late chick rearing and post-breeding periods. It has revealed substantial differences in habitat selection and utilization between the sexes and breeding stages of CIFB.

### Brooding period

During the brooding period, about May and June, when both males and females attend and provision their chicks regularly, the foraging behaviour of the sexes differed and changed depending on the year. In 2009, a year with average marine productivity and hence supposedly average prey availability, both sexes made foraging trips of similar duration, range and covered distance,. Even though males flew farther north towards waters of south-west Java than females, both sexes foraged mainly over deep, oceanic waters in a radius of about 200 km around CI. During that time, the chlorophyll *a* concentrations of those foraging areas were average with regard to the six year study period and the foraging habitat represented typical tropical oceanic waters which are generally characterised by low prey availability [36,37]. Thus, bi-parental chick provisioning during that time was probably crucial to provision the chick sufficiently under these conditions. The utilised habitat is similar to that of Great Frigatebirds breeding on Europa Island and Aldabra Island in the eastern Indian Ocean where both sexes forage mainly in oceanic waters around their respective colonies [25,27,28].

In 2010, chlorophyll *a* concentrations around CI were lower than the six year average. Female foraging behaviours were similar to 2009, but males undertook longer and farther trips to more northern marine habitats. Males now utilised foraging areas including both oceanic waters and costal waters off southern Java, particularly Java Head. The marine conditions around Java are different from those farther south around CI. They are relatively shallow and have higher chlorophyll *a* concentrations and hence most likely higher prey availability than the oceanic waters around the breeding island where females continued to forage. Those foraging areas more closely match those of the Magnificent Frigatebird that generally forages in coastal waters [9,12,26].

Strong sexual segregation in foraging habitat has been found in various seabird species and is assumed to function to reduce intra-specific competition for prey [19,21,38]. Stable Isotope Analyses on the blood of brooding CIFB revealed that both sexes feed on the same trophic level during this breeding stage (Hennicke et al., unpubl. data). Under the regular marine conditions of 2009, intra-specific competition did supposedly not play a large enough role to cause sex differences in foraging behaviour and strong spatial segregation. However, under the less productive conditions of 2010, the intra-specific competition for prey may have been increased leading to movements of the males farther to the north to more productive waters.

The reason why males instead of females changed their foraging behaviour in the less productive year might be related to the pronounced reversed sexual size dimorphism. In size dimorphic species, the smaller sex generally has less body reserves and is hence less able to cope with decreased prey availability [19,39]. Therefore, when conditions deteriorate, males would leave for more productive marine areas before females would, even though travelling farther would increase their foraging cost and potentially even compromise their reproductive success. While the CIFB males foraged in more productive waters, their chick provisioning frequency was about four times less than in 2009 due to the longer trip durations in 2010, suggesting a reduction of the investment into the chicks. In long-lived species like frigatebirds, investment into survival (i.e. future reproduction) is considered to be more adaptive than investment into current offspring [40].

### Late chick rearing period

Once the chicks no longer required constant attendance at the nest, female foraging behaviours changed. Throughout the late chick rearing period (i.e. with increasing chick age), females increased their foraging range and foraging trips got progressively longer. On average the trip duration was twice as long as during the brooding period. Consequently, chick provisioning by females decreased substantially to approximately every 5 days, in contrast to every 2–3 days during brooding. In addition, females also foraged in more northern areas and started to exploit the rich coastal waters of southern Java, where only males had foraged during the brooding period. Thus, females progressively occupied the niche used by males previously by exploiting more distant areas of high productivity.

CI is exposed to the cold and productive waters of the Java Trench upwelling along the south coast of Java from about July to October [29]. This provides, for oceanic waters, relatively high chlorophyll *a* concentrations close to the island, particularly during August and September when males reduce their breeding investment. In October, the productive waters recede northwards and hence prey abundance around CI is likely to decrease. However, by this time chicks are large enough to be left alone on the nest for increasing time periods and to sustain longer fasts between two provisioning visits. Thus, the females can fly farther away to exploit the more distant but also more nutrient-rich waters, such as off the Java Coast, and by doing so provision their chicks sufficiently with less need for provisioning by males. Apparently prey availability in the more distant waters exploited by the females later in the chick rearing season is high enough to compensate for the decreased chick provisioning rates caused by longer foraging trips and reduced support of their partners.

### Post-breeding period

When the females finished their breeding activity, they flew north to equatorial waters of the South China Sea and the Java Sea where they roosted on small islets off the coasts of Borneo, Sumatra and the Malay Peninsula, locations similar to where they have been observed roosting previously [41,42]. All individuals remained at their respective roosting sites for the whole period they were tracked, about 2–5 months, until transmissions ceased due to battery exhaustion.

There, the birds showed typical central place foraging behaviour, departing from and returning to the same location [43], even though they were not constrained in their foraging movements by incubation or chick provisioning. In addition, the foraging trips from the roosts were during daylight hours only and thus were limited to short distances. This suggests very good prey availability in those marine areas. The seas of the Sunda Shelf surrounding the roost islands are relatively shallow [44] and the chlorophyll *a* concentrations are substantially higher compared to the waters around CI. The high freshwater input from equatorial rivers probably increases productivity substantially over oceanic waters, and the shallow water depths might result in high prey accessibility without the need for subsurface predators which are more abundant in oceanic waters [45]. In addition, CIFB might also forage on other prey items during that time, as has been observed in other frigatebird species [14,46].

None of the females had travelled back to CI by April/May when the batteries of the satellite tags were finally exhausted. Even if the females had returned to CI immediately after the tags had stopped, it would have been too late for them to engage in a breeding attempt during this season as CIFB breeding activity starts around February to March [10,47]. Thus, the females were obligated to skip a breeding season and they potentially stay in the productive Sunda Shelf waters until the next season begins. This finding strongly supports a biennial breeding cycle for females which was suggested by Nelson [3,10] for CIFB females that raise their chick to independence for up to 15 months after hatching.

In contrast, the male that was tracked through his post-breeding season left his roost in January and returned to CI. Thus, he arrived in time to participate in the breeding season only one year after his last breeding attempt (when he was equipped with the satellite tag). It is not known if he actually engaged in breeding, but, although based on only a single observation and larger sample sizes for both males and females are required for confirmation, this finding shows for the first time that male and female CIFB potentially have different breeding cycles.
